# A Substitute for Gum Arabic

**Published:** 1855-03

**Authors:** Geo. G. Shumard

**Affiliations:** Fort Smith, Ark.


GUM MEZQUITE AS A SUBSTITUTE FOR GUM ARABIC.—BY GEO. G. SHUMARD, M. D.—Fort Smith, Ark.—This gum (for which I propose the name of *Gum Mezquite*,) is believed to occur in inexhaustible quantities, and will no doubt hereafter prove a valuable source of revenue to the State of Texas, New Mexico, and the adjacent Indian territory, besides affording employment to the different tribes of Indians now roving on the plains, many of whom would no doubt be glad to gather and deliver it to the frontier posts for a very small compensation.

The *Mezquite Tree*, from which the gum is obtained, is by far the most abundant tree of the plains, covering thousands of miles of surface, and always flourishes most luxuriantly in elevated and dry regions. The gum exudes spontaneously in a semi-fluid state from the bark of the trunk and branches, and soon hardens by exposure to the atmosphere, forming more or less rounded and variously colored masses, weighing each from a few grains to several ounces. These soon bleach and whiten upon exposure to the light of the sun, finally becoming nearly colorless, semi-transparent, and often filled with minute fissures. Specimens collected from the trunks of the trees were generally found to be less pure and more highly colored than when obtained from the branches. The gum may be collected during the months of July, August and September, but the most favorable period for that purpose is in the latter part of August, when it may be obtained in the greatest abundance and with but little trouble. The quantity yielded by each tree, varies from an ounce to three pounds, but incisions made in the bark not only greatly facilitate its exudation, but cause the tree to yield a much greater amount. As it is, a good collector would probably be able to gather from ten to twenty pounds a day; were incisions resorted to, probably double the amount might be obtained. —*Western Med. Jour.*

